# The association of *Chlamydia trachomatis* and *Mycoplasma genitalium* infection with the vaginal metabolome

**DOI:** 10.1038/s41598-020-60179-z

**Published:** 2020-02-25

**Authors:** Joanna-Lynn C. Borgogna, Michelle D. Shardell, Carl J. Yeoman, Khalil G. Ghanem, Herlin Kadriu, Alexander V. Ulanov, Charlotte A. Gaydos, Justin Hardick, Courtney K. Robinson, Patrik M. Bavoil, Jacques Ravel, Rebecca M. Brotman, Susan Tuddenham

**Affiliations:** 1 Department of Microbiology and Immunology, Montana State University, Bozeman, MT USA; 2 Department of Epidemiology and Public Health, University of Maryland School of Medicine, Baltimore, MD USA; 3 Institute for Genome Sciences, University of Maryland School of Medicine, Baltimore, MD USA; 4 Department of Animal and Range Sciences, Montana State University, Bozeman, MT USA; 5 Department of Medicine, Johns Hopkins University School of Medicine, Baltimore, MD USA; 6 Roy J. Carver Biotechnology Center, University of Illinois, Urbana-Champaign, IL USA; 7 Department of Microbial Pathogenesis, University of Maryland School of Medicine, Baltimore, MD USA; 8 Department of Microbiology and Immunology, University of Maryland School of Medicine, Baltimore, MD USA

**Keywords:** Metabolomics, Infectious-disease diagnostics

## Abstract

*Chlamydia trachomatis* (CT) and *Mycoplasma genitalium* (MG) are two highly prevalent bacterial sexually transmitted infections (STIs) with a significant rate of co-infection in some populations. Vaginal metabolites are influenced by resident vaginal microbiota, affect susceptibility to sexually transmitted infections (STIs), and may impact local inflammation and patient symptoms. Examining the vaginal metabolome in the context of CT mono (CT+) and CT/MG co-infection (CT+/MG+) may identify biomarkers for infection or provide new insights into disease etiology and pathogenesis. Yet, the vaginal metabolome in the setting of CT infection is understudied and the composition of the vaginal metabolome in CT/MG co-infected women is unknown. Therefore, in this analysis, we used an untargeted metabolomic approach combined with 16S rRNA gene amplicon sequencing to characterize the vaginal microbiota and metabolomes of CT+, CT+/MG+, and uninfected women. We found that CT+ and CT+/MG+ women had distinct vaginal metabolomic profiles as compared to uninfected women both before and after adjustment for the vaginal microbiota. This study provides important foundational data documenting differences in the vaginal metabolome between CT+, CT+/MG+ and uninfected women. These data may guide future mechanistic studies that seek to provide insight into the pathogenesis of CT and CT/MG infections.

## Introduction

Vaginal metabolites are influenced by resident vaginal microbiota, affect susceptibility to sexually transmitted infections (STIs), and impact local inflammation and symptoms^1,2^. An “optimal” vaginal microbiota is dominated by *Lactobacillus* species, which produce the metabolite lactic acid. Lactic acid lowers the pH of the vaginal microenvironment^1–4^, and, through immunomodulatory and direct inhibitory effects, may protect against acquisition of STIs, including *Chlamydia trachomatis* (CT) and HIV^2,5–7^. In contrast, women with non-optimal microbiota, as epitomized by the clinical condition of bacterial vaginosis (BV), have vaginal microbial communities that are low in *Lactobacillus* spp.; and are instead colonized by a variety of anaerobes which generally produce very little or no lactic acid. Some of these bacteria produce metabolites such as biogenic amines and short chain fatty acids that may be pro-inflammatory and have been linked with symptoms such as vaginal malodor and discomfort^2,8–12^. These metabolites may also increase susceptibility to STIs, including bolstering chlamydial persistence^8,13–16^. Women with low*-Lactobacillus* non-optimal vaginal microbiota have an increased risk for acquisition of STIs and ascending infection (including pelvic inflammatory disease [PID])^17–21^.

CT is the most prevalent bacterial STI in the United States, and causes significant morbidity, including PID and infertility in young women^22^. *Mycoplasma genitalium* (MG) is an emerging, highly drug resistant STI, which appears to be more prevalent than gonorrhea, and has been linked to cervicitis and PID in women^23–25^, though current CDC guidelines recommend consideration for testing and treatment only in women with persistent cervicitis^26^.

Interestingly, rates of co-infection with CT and MG are high in many female populations (36% of CT infected women had MG co-infection in one study done in Baltimore, MD,^27^, nearly 38% of CT infected women were found to have MG co-infection in a second study^28^ and 70.7% of women with MG in a high risk cohort were found to have CT co-infection^25^. Endometrial MG infection has been associated with endometritis and endometrial CT infection, suggesting that these infections may co-locate or perhaps affect PID risk in some women^25^; however the impact of co-infection on patient outcomes is understudied^1^.

New molecular techniques have enabled a higher resolution understanding of BV, the vaginal microbiota, and associations with STI, including CT and MG infections. 16S rRNA gene amplicon sequencing techniques have identified several different kinds vaginal microbiota, or “community state types” (CSTs)^29^ based on bacterial composition and relative abundance. Four are dominated by different *Lactobacillus* species (CST I, *Lactobacillus crispatus*; CST II, *L. gasseri*; CST III, *L. iners*; CST V, *L. jensenii*). A fifth community (CST IV) is low in *Lactobacillus* spp. and has been termed “molecular-Bacterial Vaginosis” (molecular-BV)^3^. CST IV and CST III have been associated with increased risk of CT acquisition^30^. Emerging evidence suggests that low-*Lactobacillus* vaginal microbiota may be associated with increased risk for MG infection as well^18,30^.

Associations between the vaginal microbiota and enhanced risk of CT and MG infection are likely mediated, at least in part, through vaginal metabolites. CT and MG may also independently influence the vaginal metabolome through direct, host-mediated or microbiota-mediated mechanisms, perhaps impacting clinical outcomes. Therefore, examining the vaginal metabolome in the context of these infections may identify biomarkers for infection or provide new insights into disease etiology and pathogenesis. Yet, to date, only one study has examined the vaginal metabolome in the setting of CT infection^31^, and the composition of the vaginal metabolome in CT/MG co-infected women is unknown. Therefore, in this study, we utilized untargeted gas chromatography-mass spectrometry (GC-MS) to characterize the vaginal metabolome of CT mono-, CT/MG co-infected, and uninfected women. As STI-infected women are known to have a higher prevalence of BV^30,32,33^, we additionally integrated analysis of the vaginal metabolome with 16S rRNA gene amplicon sequencing in order to understand whether the identified differences in vaginal metabolites remained statistically significant after controlling for the composition of the vaginal microbiota.

## Results

### Participant characteristics and vaginal microbiota composition (CST) by infection state

Vaginal samples from 145 race-matched women (77 uninfected, 54 CT+ (mono-infected), and 14 CT+/MG+ (co-infected)) were analyzed. CT+ and CT+/MG+ women were generally younger, had more sexual partners, and a lower proportion reported “always” using condoms as compared to uninfected women (Supplementary Table S1). Seven (50%) of the CT+/MG+ women had evidence of macrolide resistance based on the SpeeDx MG testing. A much larger proportion of CT+ (64.8%) and CT+/MG+ (78.6%) women had CST IV (low-*Lactobacillus* species vaginal microbiota) as compared with uninfected women (26.0%, p < 0.01), and Supplementary Fig. S1). The majority of women (45 of the 54 CT mono-infected women (83%) and 12 of the 14 CT+/MG+ co-infected women (85%)) reported one or more symptom or were diagnosed with pelvic inflammatory disease on exam.

### Vaginal metabolomic profiles distinguish women based on infection status and CST

**Figure 1:**
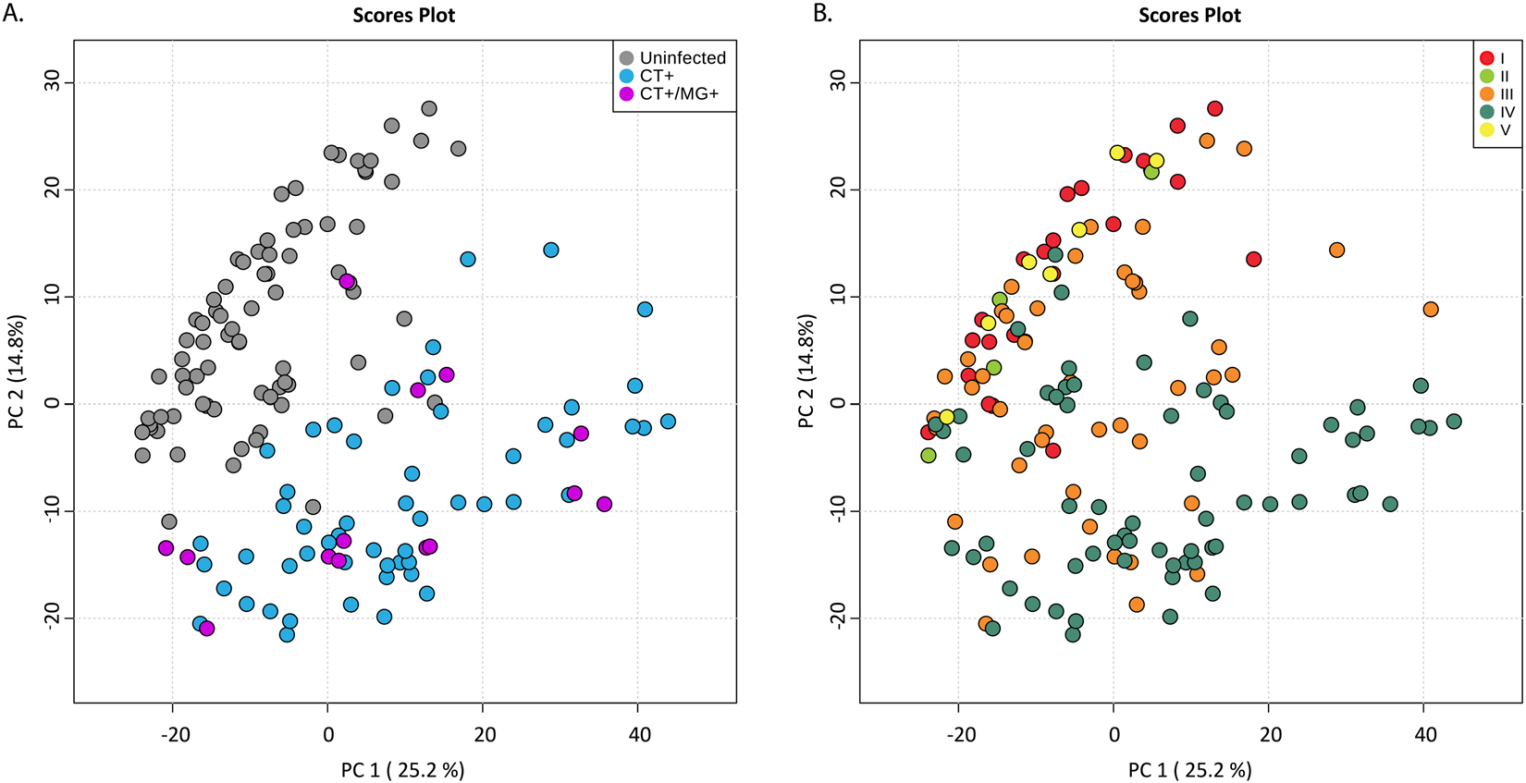
The vaginal metabolome clusters by infection status and by CST. Two-dimensional principal component analysis (PCA) score plots demonstrate statistical clustering of metabolites by (**A**) infection status and (**B**) by CST.

An exploratory PCA plot revealed that metabolomes clustered by infection status with infected women clustering separately from uninfected women, and CT+ and CT+/MG+ women largely clustering together (Fig. 1A). A second PCA showed that women in CST IV (low-*Lactobacillus* vaginal microbiota) clustered separately from those with *L. crispatus*-, *L. gasseri*-, or *L. jensenii*- dominated vaginal microbiota (CST I, II, and V, respectively). Women with *L. iners* dominated vaginal microbiota (CST III) were interspersed between the two clusters (Fig. 1B). Both multiple linear regression and PLS-DA identified several metabolites that significantly distinguished: (1) CT+ vs. uninfected women; (2) CT+/MG+ vs. uninfected women; and 3) CT+/MG+ vs. CT+ women (Supplementary Tables S2 and S3). Given the demographic and behavioral differences between cases and controls (Supplementary Table S1), we also assessed the potential impact of these factors upon the vaginal metabolome. Results from this analysis indicate that age, number of recent sexual partners, and condom use, among others, were not significantly associated with the vaginal metabolome. (See Supplementary Table S4). The only significant variables that contributed >3% of the variation to the vaginal metabolome were infection status (12%) and CST (10%).

### Multiple vaginal metabolites distinguish CT mono-, CT/MG co-infected and uninfected women before and after adjustment for CST

Multiple linear regression demonstrated that there were significant differences in individual vaginal metabolites in CT+ and CT+/MG+ compared with uninfected women both before and after adjustment for CST. Supplementary Tables S2 and S3 summarize all significant metabolites identified by multiple linear regression, grouped by metabolic pathway based on the Kyoto Encyclopedia of Genes and Genomes (KEGG). All results reported below have a q-value <0.05 unless otherwise stated.

In unadjusted analyses, the relative concentrations of 99 and 64 metabolites were significantly different when comparing CT+ and CT+/MG+ women to uninfected women, respectively (Supplementary Table S2). After adjustment for CST, 64 and 36 metabolites significantly differed when comparing CT+ and CT+/MG+ co-infected women, respectively, to uninfected women (Supplementary Table S3). 93% of metabolites CT+ vs. uninfected patients (60/64) and 94% of metabolites in CT+/MG+ co-infected women vs. uninfected women (34/36)) which were significant before adjustment for CST were also significant after adjustment for CST. Comparatively fewer metabolites differentiated CT+/MG+ and CT+ women both before (7 metabolites) and after adjustment for CST (3 metabolites). All of the metabolites that distinguished these two groups after adjustment for CST were significant prior to adjustment for CST.

#### Amino acid metabolism

Women with CST IV irrespective of infection status, had greater vaginal concentrations of the biogenic amines cadaverine, putrescine, tyramine and ethanolamine (part of the lipid super pathway but is also considered a biogenic amine) based on linear regression (one-sided p-value <0.05) compared with women with *Lactobacillus*-dominated CSTs. Consistent with this result, (given the higher proportion of women with CST IV microbiota in the infected groups), before adjustment for CST, the biogenic amines cadaverine, putrescine, tyramine and ethanolamine were increased in the CT+, and cadaverine, putrescine and ethanolamine were increased in the CT+/MG+ as compared with the uninfected women. After adjustment for CST, only ethanolamine was increased in infected as compared with uninfected patients.

Cinnamic acid or its associated derivatives were higher in CT+ and CT+/MG+ women compared to uninfected women with and without adjustment for CST. Hydrocinnamic acid was increased in CT+/MG+ as compared to CT+ women, but there were no differences in biogenic amines either before or after adjustment for CST between these two groups.

#### Carbohydrate metabolism

Several sugars and derivatives, including lactose and sorbitol, were significantly lower in both CT+ and CT+/MG+ women compared to uninfected women, irrespective of adjustment for CST. Metabolites involved in pentose metabolism, such as xylose, were higher in CT+ and CT+/MG+ relative to uninfected women both before and after adjustment for CST. Glyceric acid, which is involved in glycolysis, was decreased in CT+ and CT+/MG+ relative to uninfected women before and after adjustment for CST.

#### Lipid metabolism

Multiple long chain fatty acids (LCFAs), including decanoic, pentadecanoic, heptadecanoic, oleic, arachidic, behenic, cerotic, and myristic acid were higher in CT+ as compared to uninfected women without adjustment for CST; the majority of these remained significantly higher after adjustment for CST. Fewer LCFAs, including pentadecanoic, heptadecanoic, and oleic acids were elevated in CT+/MG+ as compared to uninfected women, all of which remained significantly higher after adjustment for CST. The LCFA palmitoleic acid was decreased in CT+/MG+ as compared to CT+ women, but only before adjustment for CST. The sterol, 5α-cholestan-3-ol, was higher in CT mono-infected women compared to uninfected women without adjustment for CST. Multiple long chain fatty alcohols including octadecanol, eicosanol, heptadecanol and hexadecanol, were higher with and without CST adjustment in CT+ and CT+/MG+ as compared with uninfected women. Octadecanol was lower in CT+/MG+ co-infected as compared with CT+ women both before and after adjustment for CST.

#### Energy metabolism

α-ketoglutaric acid, citric acid and glutaric acid, all metabolites involved in the TCA cycle, were lower in CT+ compared to uninfected women both before and after adjustment for CST. These metabolites were also significantly decreased in CT+/MG+ women relative to uninfected women after adjustment for CST.

#### Nucleic acid metabolism

Several metabolites involved in purine and pyrimidine metabolism, including adenine, guanine and cytosine, were increased in CT+ relative to uninfected women both before and after adjustment for CST. These were also higher in CT/MG co-infected women before adjustment for CST; guanine and cytosine were higher after adjustment for CST. When comparing CT+/MG+ to CT+ women, we did not observe any significant differences in nucleotide abundance with or without adjustment for CST.

### Partial least squares discriminate analysis (PLS-DA)

**Figure 2:**
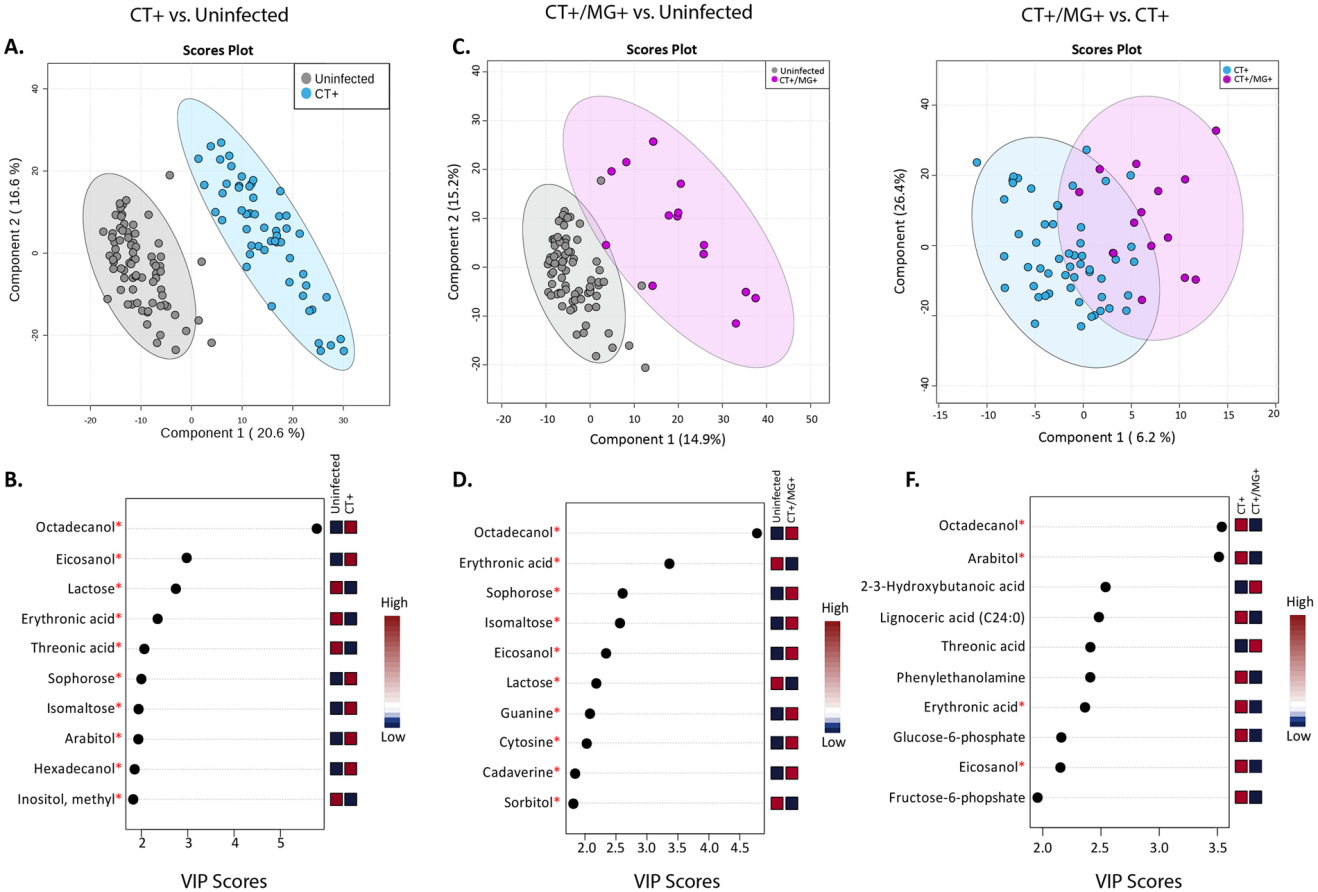
Discriminatory metabolites associated with infection status. CT+ and uninfected women are compared in the first column (**A,B**), CT+/MG+ and uninfected women are compared in second column (**C,D**) and CT+/MG+ women compared to CT+ women are compared in the last row (**E,F**). (**A,C,E**) Show discrimination of groups using partial least squares discriminant analysis. (**B,D,F**) show the metabolites most strongly influencing discrimination by the PLS-DA. The variable importance in projection (VIP) score is the weighted sum of squares for the PLS-DA loading with the amount of variation explained by each component taken into account. Asterisks indicate metabolites which are also significant via regression analyses.

We utilized PLS-DA to identify the most discriminatory metabolites between each infection state (Fig. 2). PLS-DA was able to effectively separate CT+ and CT+/MG+ from uninfected women in two components with accuracies ranging from 94–98%. Permutation tests provided evidence that the differences between these clusters were significant (CT+ vs. uninfected women, p-value <0.0001, 0/2000 permutations; CT+/MG+ vs. uninfected women, p-value =0.0025, 5/2000 permutations). Variable importance plots (VIP) indicated that octadecanol, eicosanol, and lactose (VIP scores> 2.5) contributed the most to the class separation between CT+ and uninfected women, with octadecanol and eicosanol being higher in CT+ women and lactose being lower in CT+ than uninfected women. (Fig. 2B). The most important discriminatory metabolites between CT+/MG+ and uninfected women were octadecanol, erythronic acid, sophorose, and isomaltose with octadecanol, sophorose and isomaltose being higher in CT+/MG+ than uninfected women. (Fig. 2D). PLS-DA was not able to effectively separate CT+/MG+ co-infected women from CT+ women (Fig. 2E). The lack of separation was cross-validated with R^2^ coefficients of 0.39 and 0.79 and Q^2^ values of −0.35 and 0.22. Permutation tests further provided evidence against the separation of these two groups (p-value 0.167, 334/2000 permutations).

**Figure 3:**
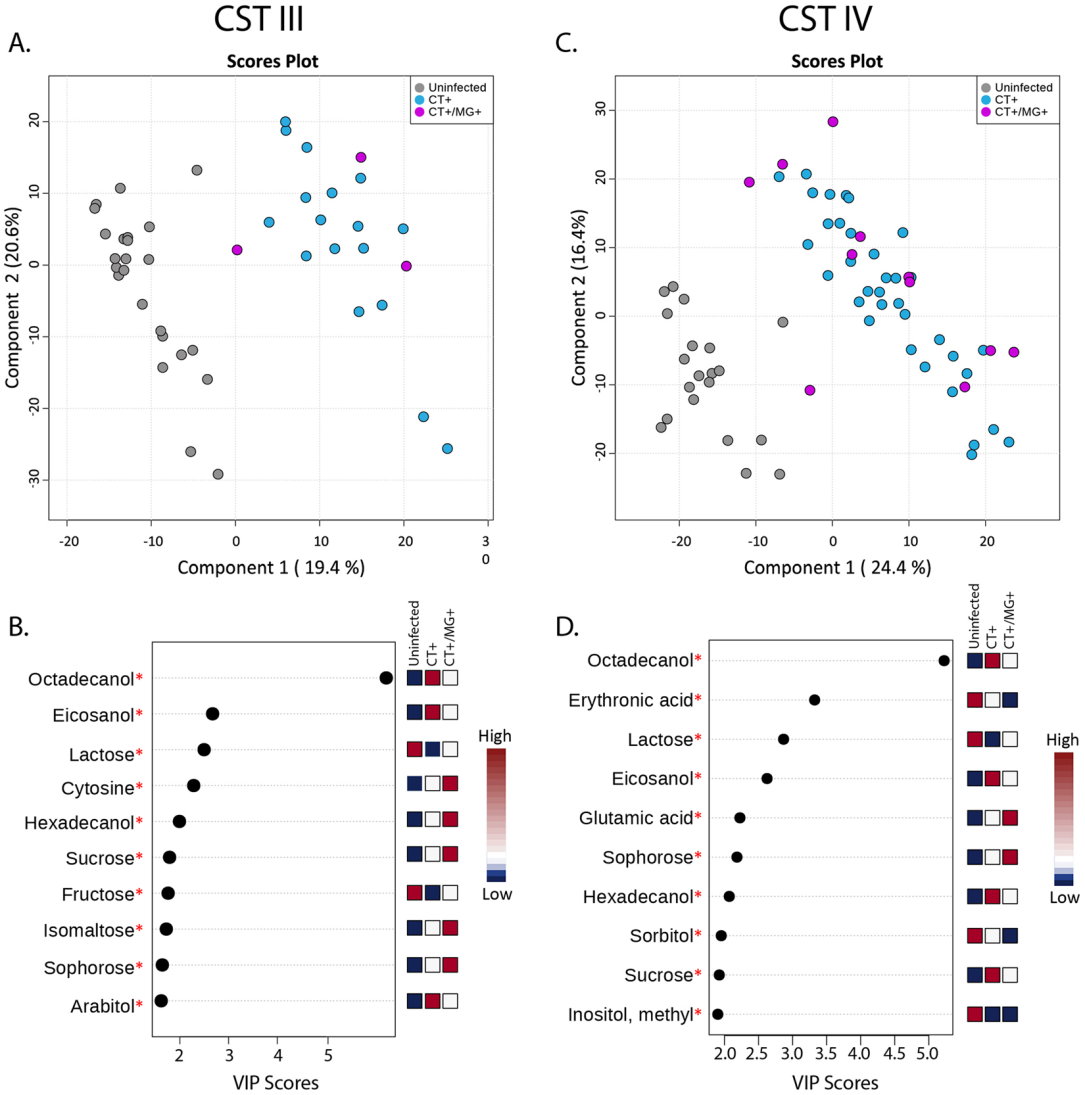
Discriminatory metabolites associated with infection status stratified by CST. Women within CST-III are compared in the first column (**A,B**) and women within CST-IV are compared in the second column (**C,D**). (**A,C**) Show discrimination of groups using partial least squares discriminant analysis. (**B,D**) Show the metabolites most strongly influencing discrimination by the PLS-DA. Asterisks indicate metabolites which are also significant via regression analyses.

Finally, we subset the data into CST III (*L. iners* dominated) and CST-IV (low-*Lactobacillus*) and performed separate PLS-DAs to evaluate differences between infection status within strata of CST (Fig. 3). CT+ and CT+/MG+ women clustered together and were discriminated from uninfected women, irrespective of CST (CST III, p-value = <0.0001, 0/2000 permutations; CST IV p-value <0.0001, 0/2000 permutations). Octadecanol, eicosanol and lactose were significant in discriminating between the CT+ and uninfected subjects for women in both CST III and CST IV.

## Discussion

We found that numerous vaginal metabolites distinguished CT+ and CT+/MG+ from uninfected women, with and without adjustment for the composition of the vaginal microbiota. When comparing CT+/MG+ to CT+ women, we observed significant overlap of vaginal metabolome profiles.

Previous literature suggests that women with BV are at increased risk for acquisition of CT and MG^1^, and women with CT infection are most likely to be in CST IV^30^. Consistent with this, a larger proportion of the CT+ and CT+/MG+ as compared with the uninfected women in our study cohort had CST IV microbiota. Since biogenic amines (thought to be produced by BV-associated bacteria) are known to be higher in women with CST IV^8^, it is not surprising, therefore, that the biogenic amines putrescine and cadaverine were higher in CT mono- and CT/MG co-infected as compared to uninfected women prior to, but not after adjustment for CST. Biogenic amine production involves specific amino acid decarboxylation reactions, and is a well-described bacterial acid resistance and mitigation mechanism^8,16,32,34,35^. This reduction of vaginal acidity may promote colonization by other BV-associated bacteria or further overgrowth of existing BV-associated bacteria. Additionally, the production of biogenic amines may increase the virulence of various pathogens, and may protect pathogens from innate immune defenses^36,37^. Biogenic amines may reduce the host’s immune defenses against STIs, including CT or MG^8^.

While some of the differences in vaginal metabolites between infection groups (such as putrescine and cadaverine, above) may relate to the higher proportion of infected women with CST IV, we cannot discount the possibility that these CST-related metabolites are important to the infection process. Differences in other metabolites remained consistent even after adjustment for CST, suggesting a role for the infecting pathogen(s) (or perhaps an interaction between the host, pathogen and underlying microbiota) in impacting the metabolome. For example, we observed significantly higher concentrations of most LCFAs and fatty alcohols when comparing the vaginal metabolites of CT+ and CT+/MG+ to uninfected women both before and after adjustment for CST. This effect, however, appeared to be attenuated by MG co-infection: CT+/MG+ women had several LCFAs and fatty alcohols that were lower compared to CT+ women.

It is possible that the higher levels of LCFAs are related to CT survival, growth, and/or pathogenesis. Chlamydiae are obligate intracellular organisms that are auxotrophic for a variety of essential metabolites, including many lipids that they acquire from the host cell. Lipids are trafficked from various sources within the host cell into the inclusion and to the envelope of the reticulate bodies. The acquisition of lipids from host stores is thought to be critical for chlamydial replication and development^38^. In eukaryotic cells, cholesterol esters, which are formed by the addition of a LCFA to the hydroxyl group of cholesterol, are the main transport and storage form of cholesterol^39^. The enzyme acyl-CoA:cholesterol acyltransferase (ACAT) catalyzes the production of these esters^40^. This process is important because in the un-esterified form, cholesterol can be toxic to the cell. A recent report^40^ showed that chlamydial growth can be inhibited by blocking ACAT with a specific inhibitor, 4-hydroxycinnamic acid amide. Addition of a cholesterol ester (cholesterol esterified with a LCFA) rescued ACAT dependent inhibition^40^.

These data raise the question of whether CT may be upregulating host production of LCFAs and cholesterol esters as part of a survival or growth mechanism. The relatively lower levels of lipid and lipid derivatives in CT/MG co-infected women versus uninfected women may reflect a direct or indirect competition between CT and MG for host lipids. Alternatively, the higher fatty alcohols and LCFAs may reflect greater wax ester production. Wax esters, a type of neutral lipid, are formed by the esterification of a fatty alcohol with a fatty acid derived from either acyl-CoA or acyl-ACP^41^. Neutral lipids are commonly found in vaginal fluid, along with secretions from the cervix, squamous epithelial cells and material from the sebaceous, apocrine and Bartholin’s glands^42^. Thus, it is possible that the higher abundance of fatty acids, LCFAs and fatty alcohols reflects greater vaginal discharge associated with chlamydial infections, whether due to increased polymorphonuclear leukocytes or increased secretions from host cells. These questions are intriguing, but speculative. Larger, mechanistic studies will be required to further explore them.

No studies have examined the vaginal metabolome in the context of MG mono- or CT/MG co-infection, and only one other report^31^ has examined the vaginal metabolome in the context of CT infection. That study found fewer distinguishing metabolites but had a number of important differences from ours, including a smaller sample size, the use of 1H-NMR rather than GC-MS to detect a total of only 54 metabolites and a lack of testing for MG. Furthermore, the study design was quite different: CT infected women in the Parolin study were specifically selected not to have BV. Importantly, by controlling for CST rather than eliminating subjects with CST IV microbiota, our results may capture important changes that occur as a result of complex interactions between the host, CT and dysbiotic vaginal microbiota which would be missed by an approach that excludes CT mono-infected women with BV.

There are several limitations to our study. First, while we have the largest group of CT+ women studied to date in a metabolomic analysis, the CT+/MG+ co-infected group was small, limiting our ability to control for other potential confounders^8^. Secondly, these data are cross-sectional, with samples taken at the time of CT and CT/MG infection. Therefore, causal inferences (e.g. regarding the impact of the vaginal metabolome on susceptibility to CT or CT/MG co-infection) are not possible; nor is it possible to account for short term fluctuations in the vaginal microbiota. We do not have information on whether these represented first infections or re-infections. Cases were younger, less likely to use condoms, and reported more recent sexual partners than controls. While these factors were not found in univariate analysis to influence our outcome (i.e. the vaginal metabolome), we cannot completely exclude the possibility that these differences (or other unmeasured factors, such as *Candida* colonization) may impact the relationship between infection status and the metabolome. A large number of samples had to be excluded due to insufficient sample for metabolomics testing, unavailable 16S rRNA gene sequencing data or co-infection with *N. gonorrhoeae* or *Trichomonas vaginalis*, and there may have been unmeasured differences in these excluded samples. Batch effects could have introduced bias. Although we took a broad, untargeted GC-MS based approach, the library of identified metabolites does not reflect all the small metabolites present within the vaginal environment, and we may have missed crucial metabolites in our analysis. Importantly, the use of untargeted GC-based mass spectrometry excluded the detection of volatile fatty acids such as trimethylamine, acetate, or butyrate. The vaginal samples were stored in Amies transport medium. A number of metabolites, including lactic acid, were found in the transport medium and therefore had to be removed from analysis. Given limited sample size, we were not able to assess associations between relative abundance of specific bacterial species and vaginal metabolites. Finally, given the limitations of study design, we are only able to identify broad associations - it is not possible to untangle from this data what specific factors, or interaction between factors (i.e. host, vaginal microbiota and pathogen) are impacting metabolites and ultimately how this may affect susceptibility to, clearance of, or morbidity from CT and MG infections.

Despite these limitations, our study shows that a distinct vaginal metabolic profile distinguishes CT+ and CT+/MG+ from uninfected women, with significant overlap between CT+ and CT+/MG+ women. This study provides important foundational data documenting cross-sectional differences in the vaginal metabolome. Larger, mechanistic studies will be needed to explore the complex interactions between host, vaginal microbiota and pathogens, in order to better understand the pathogenesis of CT and CT/MG co-infection.

## Methods

### Study population and sample collection

Patient samples were drawn from three separate cohorts. CT positive cases (CT+) were drawn from the first (CT infected) visit of the Chlamydia Adolescent/Adult Reproductive Management (CHARM) study^43^. CHARM recruited predominantly African-American CT+ young women of reproductive age in Baltimore, MD and followed them over time after treatment for CT. Women were also tested for gonorrhea (GC) via NAAT and trichomoniasis via wet mount microscopy. Race-matched uninfected controls were drawn from enrollment visits of the Hormonal Contraception Longitudinal (HCL) study, (a cohort of low-risk women of reproductive age starting and stopping hormonal contraception recruited in Baltimore, MD^44^) and the Human Microbiome Project Prospective Women’s Health study at UMB (UMB-HMP), (a cohort of women of reproductive age recruited from clinics in Alabama and followed daily for 10 weeks)^45^. Women in HCL and UMB-HMP were tested for CT, GC (via NAAT). Trichomoniasis was tested for via microscopy in HCL and inPouch in UMB-HMP. In UMB-HMP, if diagnosis of an STI or symptomatic condition was made at baseline, it was treated, and subjects were offered enrollment 30 days after treatment completion. Subjects were tested again for gonorrhea, chlamydia and trichomoniasis at their enrollment visit. All samples were self-collected mid-vaginal Copan Eswab specimens placed in Amies transport media and frozen at −80 °C until use.

### Ethics statement

All participants provided written informed consent. Ethical approval was obtained from the Institutional Review Boards of Johns Hopkins University, the University of Maryland Baltimore (UMB), and Montana State University, and all research was conducted in compliance with relevant guidelines and regulations.

#### Taxonomic assignment, community state type profiling and *M. genitalium* detection

Genomic DNA was extracted from 300 µl aliquots of re-suspended Amies solution as previously reported for the respective papers: (CT+ and CT+/MG+ cases^46^), (controls recruited from Alabama^47^), and (controls recruited from Maryland^44^). For the QS DSP Midi kit extraction, samples were thawed on ice and a 500 µl aliquot from the vaginal swab was used as input^48^. For the MagAttract kit, samples were thawed on ice and a 200 µl aliquot from the vaginal swab was used as input following the manufacturer protocol. Cells were lysed via shaking with 1.0 mm glass beads on the TissueLyser (Qiagen) at 20 Hz for 20 minutes and the final elution volume was 110 µl. Negative controls of water were extracted in the same manner as samples.

The vaginal microbiota was characterized by sequencing the V3-V4 regions of the 16S rRNA gene. Library construction was performed using either a 1-step protocol with sequencing carried out on the Illumina MiSeq platform for 600 cycles, resulting in 2 ×300 bp read lengths, or a 2-step PCR protocol, sequencing was carried out on the Illumina HiSeq. 2500 platform using Rapid Run Chemistry, and sequence data were processed according to Holm *et al*.^48^. Amplicon sequence variants (ASVs) generated by DADA2 were classified taxonomically using the RDP Naïve Bayesian Classifier^49^ trained with the SILVA v128 16S rRNA gene database^48,50^. ASVs of major vaginal taxa were assigned species-level annotations using speciateIT (http://ravel-lab.org/speciateit/). Taxonomic data from a pool of over 12,000 vaginal samples were used to assign community state types (CSTs). Low-abundant taxa present at less than 10^−5.5^ abundance across the project were removed and samples with fewer than 5,000 reads were removed from analysis. Hierarchical clustering based on Jensen-Shannon distances between samples and Ward linkage was used to determine clusters for CST assignment. Four CSTs dominated by *Lactobacillus* spp. were identified: CST I (*L. crispatus*), CST II (*L. gasseri*), CST III (*L. iners*), and CST V (*L. jensenii*)^51^. CST IV contained more diverse organisms with low levels of *Lactobacillus* spp., (See Supplementary Fig. S1 for heatmap of 25 most abundant taxa with CST groupings).

Finally, DNA extracted from vaginal swabs was tested for MG using SpeeDx, a multiplex qPCR DNA based assay for detection of MG and resistance to macrolides by detecting one or more of 5 mutations in the 23 S rRNA gene^52,53^.

### Sample selection and preparation for metabolomics

289 samples were tested for MG: 157 CT+ cases from CHARM and 132 race matched CT- controls from HCL and HMP. Two control (i.e. CT-) samples tested positive for MG and were excluded. After exclusion of additional samples due to insufficient sample for metabolomic testing, unavailable 16S rRNA gene sequencing data, or co-infection with GC or *Trichomonas*, samples from 145 women were available for analysis (77 uninfected, 54 CT+ and 14 CT+/MG+ samples). N = 25 of the controls were recruited in Alabama, the remaining cases and controls were recruited from Maryland.

For quality control, an aliquot of 10 μl was taken from each sample and mixed in 10 replicates. Additionally, two samples of sterile Amies transport medium (controls) were run in duplicate. Samples, Amies controls, and QC supernatants were dried in a vacuum and derivatized with 50 μl methoxyamine hydrochloride (Sigma-Aldrich, MO, USA) (40 mg/mL^−1^ in pyridine) for 60 min at 50 °C, then with 50 μl MSTFA + 1%TMCS (Thermo, MA, USA) at 70 °C for 120 min, followed by a 2-hour incubation at room temperature. 30 μl of 1 mg/ml hentriacontanoic acid was added to each sample prior to derivatization for use as an internal standard for normalization.

Metabolite profiles were acquired using a gas-chromatography mass-spectrometry (GC-MS) system (Agilent Inc, CA, USA) consisting of an Agilent 7890 gas chromatograph, an Agilent 5975 MSD and 7683B autosampler, as previously described^54^. Briefly, gas chromatography was performed on a ZB-5MS (60 m × 0.32 mm I.D. and 0.25 mm film thickness) capillary column (Phenomenex, CA, USA). The inlet and MS interface temperatures were 250 °C, and the ion source temperature was adjusted to 230 °C. An aliquot of 1 ml was injected with the split ratio of 10:1. The helium carrier gas was kept at a constant flow rate of 2.4 ml/min. The temperature program was: 5-min isothermal heating at 70 °C, followed by an oven temperature increase of 5 °C/min to °C after which a final 10 min incubation at 310 °C was performed. The mass spectrometer was operated in positive electron impact mode (EI) at 69.9 eV ionization energy at m/z 30–800 scan range. The spectra of all chromatogram peaks were evaluated using the AMDIS 2.71 (NIST, MD, USA) using a custom-built database (460 unique metabolites) of the University of Illinois Carver Metabolomics Center. Throughout the sample preparation, data-acquisition and data-preprocessing, samples were compared to the QCs to evaluate potential variation that may have arisen in the dataset throughout the analytical study. All known artificial peaks were identified and removed prior to data mining. To allow comparison between samples, all data were normalized to the internal standard in each chromatogram and sample volume. The instrument variability was within the standard acceptance limit (5%).

### Metabolite pre-processing and statistical analyses

Metabolites detected in Amies (See Supplementary Table S5) were removed from analysis. Metabolites were excluded from analysis if they were undetected in ≥90% of samples in all biological groups (CT+, CT+/MG+ and uninfected). Remaining missing values were assumed to be below the level of detection and were imputed (across all samples, irrespective of biological groups) using one half the minimum value obtained for a given metabolite^55–58^. The final dataset available for analysis consisted of 163 metabolites. Following imputation of missing values, data were mean-centered, and a generalized log-transformation was applied to meet assumptions of linearity and normality required for regression models. Mean centering and generalized log-transformations were performed using MetaboAnalyst 4.0^58^. Metabolic super and sub pathways were identified using the Kyoto Encyclopedia of Genes and Genomes (KEGG) database.

Unadjusted associations between infection status and demographic and behavioral factors were assessed using Fisher’s exact test (Supplementary Table S1). Samples were visualized using a principal component analysis (PCA) constructed in MetaboAnalyst 4.0 (Fig. 1)^58^. A Bray-Curtis dissimilarity matrix was constructed following normalization of the 163 metabolites, and a permutational multivariate analysis of variances (PERMANOVA) was performed to evaluate the association of potential sources of variation, including demographic and behavioral factors, with the vaginal metabolome. The Bray-Curtis dissimilarity matrix and PERMANOVA were conducted using the package *vegan* in R statistical Software.

We fit a linear regression model of mean-centered, generalized log-transformed metabolites on infection status, with and without controlling for CST. For each model, a significance level $\alpha =0.05$ was used. To correct for multiple comparisons, p-values were adjusted using the Storey false discovery rate in the package *qvalue*^59^. Linear regression and false discovery rate were conducted using R Statistical Software. Fold changes (FC) were calculated using $F{C}_{i}=\exp (mean(\log ({X}_{i})-mean(\log ({Y}_{i}))$ where *i* is the metabolite of interest for the mean value of group of interest (X) and mean value of reference group (Y). Fold change values <1 indicate lower metabolite concentration group X than in group Y (e.g, CT infected versus uninfected). Metabolite fold changes were calculated for (1) CT mono-infected vs. uninfected women, (2) CT/MG co-infected vs. uninfected women, and (3) CT/MG co-infected vs. CT mono-infected women. We additionally subset the data and calculated the metabolite fold change between infection states within CST III and CST IV. CSTs I, II, and V were not included as only two CT mono-infected, and no CT/MG co-infected women had vaginal microbiota corresponding to these CSTs, limiting our ability to make comparisons to uninfected women.

We performed partial least squares discriminant analysis (PLS-DA) on the mean centered, generalized log-transformed metabolite data. PLS-DA models were assessed based upon variance (R^2^) and 10-fold cross-validation, which provides a quality assessment Q^2^ with values close to 1 indicate reliable models and negative values indicate model overfitting. To assess whether differences between the groups were significant, a permutation test using the sum of squares between/sum of squares within (B/W) ratio was calculated. Variable importance in projection (VIP) score plots were used to display the top ten discriminatory metabolites. VIP scores ≥2.5 were considered important within a given model. PLS-DA, and VIP score plots were computed using MetaboAnalyst 4.0.

## Supplementary information

Supplementary Info.

## Data Availability

The current 16S rRNA gene sequencing data for the cases can be found in SRA at: PRJNA509676. The 16S rRNA gene sequencing data for the controls recruited from Alabama can be found in SRA at: PRJNA208535. The 16S rRNA gene sequencing data for the controls recruited from Baltimore is currently in process and will be accessible through dbGaP. All data is available from the authors on reasonable request.
