# Supplementary material for: The association of Chlamydia trachomatis and Mycoplasma genitalium infection with the vaginal metabolome
Source: Sci Rep. 2020 Feb 25;10:3420. doi: 10.1038/s41598-020-60179-z (PMC7042340; doi:10.1038/s41598-020-60179-z)
Supplement: Supplementary file 1 — Supplementary Info. [file 41598_2020_60179_MOESM1_ESM.pdf]

The association of *Chlamydia trachomatis* and *Mycoplasma genitalium* infection with the vaginal metabolome

Joanna-Lynn C. Borgogna[1], Michelle D. Shardell[2,3], Carl J. Yeoman[1,4], Khalil G. Ghanem[5], Herlin Kadriu[1], Alexander V. Ulanov[6], Charlotte A. Gaydos[5], Justin Hardick[5], Courtney K. Robinson[3], Patrik M. Bavoil[7], Jacques Ravel[3,8], Rebecca M. Brotman[2,3], and Susan Tuddenham*[5]

[1] Department of Microbiology and Immunology, Montana State University, Bozeman, MT, USA

[2] Department of Epidemiology and Public Health, University of Maryland School of Medicine, Baltimore, MD, USA

[3] Institute for Genome Sciences, University of Maryland School of Medicine, Baltimore, MD, USA.

[4] Department of Animal and Range Sciences, Montana State University, Bozeman, MT, USA

[5] Department of Medicine, Johns Hopkins University School of Medicine, Baltimore, MD, USA

[6] Roy J. Carver Biotechnology Center, University of Illinois, Urbana-Champaign, IL, USA

[7] Department of Microbial Pathogenesis, University of Maryland School of Medicine, Baltimore, MD, USA.

[8] Department of Microbiology and Immunology, University of Maryland School of Medicine, Baltimore, MD, USA.

*** Correspondence:**
Susan Tuddenham

Johns Hopkins Bayview Medical Center
5200 Eastern Avenue, MFL Center Tower, Suite 381
Baltimore, MD 21224
studden1@jhmi.edu

Table S1| Demographic, behavioral, and biological factors associated with infection status

Participant Details	Uninfected (77) n (%)	CT+ (54) n (%)	CT+/MG+ (14) n (%)	P-value*
**Age**				<0.0001
13-20	7 (9.1)	39 (72.2)	10 (71.4)	
21-25	31 (40.2)	11 (20.4)	4 (28.6)	
26-30	18 (23.4)	4 (7.4)	0 (0.0)	
31-35	16 (20.8)	0 (0)	0 (0.0)	
36-45	5 (6.5)	0 (0)	0 (0.0)	
**Community State Type**				<0.0001
I	22 (28.6)	2 (3.7)	0 (0.0)	
II	4 (5.2)	0 (0.0)	0 (0.0)	
III	24 (31.2)	17 (31.5)	3 (21.4)	
IV	20 (26.0)	35 (64.8)	11 (78.6)	
V	7 (9.0)	0 (0.0)	0 (0.0)	
**Hormonal Contraceptive**				0.243
Using	28 (36.4)	21 (38.9)	2 (14.3)	
Not using	49 (63.6)	33 (61.1)	12 (85.7)	
**Sexual Partners, no (%)**				<0.0001
0 in last 2-3 months	11 (14.3)	3 (5.6)	0 (0.0)	
1 in last 2-3 months	55 (71.4)	22 (40.7)	3 (21.4)	
2 in last 2-3 months	7 (9.1)	18 (33.3)	5 (35.7)	
More than 2 in the last 2-3 months	0 (0.0)	8 (14.8)	6 (42.9)	
Declined to answer	4 (5.2)	3 (5.6)	0 (0.0)	
**Race**				0.876
African American	59 (76.6)	47 (87.0)	14 (100.0)	
Asian	2 (2.6)	1 (1.9)	0 (0.0)	
Latino	5 (6.5)	2 (3.7)	0 (0.0)	
Mixed	1 (1.3)	0 (0.0)	0 (0.0)	
Native American	1 (1.3)	0 (0.0)	0 (0.0)	
White	9 (11.7)	4 (7.4)	0 (0.0)	
**Condom Use**				<0.0001
Always	31 (40.3)	4 (7.4)	1 (7.1)	
Sometimes	7 (9.1)	37 (68.5)	12 (85.7)	
Never	25 (32.5)	13 (24.1)	1 (7.1)	
Declined to answer	14 (18.2)	0 (0.0)	0 (0.0)	
**History of Douching**				0.139
Yes	10 (13.0)	13 (24.1)	4 (28.6)	
No	67 (87.0)	41 (75.9)	10 (71.4)	
**Pelvic Inflammatory Disease**				< 0.0001
Yes	0 (0.0)	13 (24.1)	2 (14.3)	

No	77 (100.0)	41 (75.9)	12 (85.7)	
**HIV**				0.0033
Positive	0 (0.0)	3 (5.5)	2 (14.3)	
Negative	77 (100.0)	48 (88.8)	12 (85.7)	
Unknown/no answer	0 (0.0)	3 (5.5)	0 (0.0)	

* p-value determined using Fisher's exact test

Table S2| Significant metabolites associated with infection status identified by multiple linear regression and unadjusted for CST

CT+ women vs. Uninfected women							
**KEGG ID**	**Super Pathway**	**Sub Pathway**	**Metabolite**	**P-value**	**Q-value**	**Coefficient**	**Fold Change**
	Amino Acid	Alanine and Aspartate Metabolism	beta-alanine	0.0138	0.0310	0.8	2.23
C00025	Amino Acid	Glutamate Metabolism	Glutamic acid	0.0091	0.0220	1.9	7.56
C01879	Amino Acid	Glutathione Metabolism	Pyroglutamic acid	0.0073	0.0179	-0.9	0.42
C00168	Amino Acid	Glycine, Serine and Threonine Metabolism	3-Hydroxypyruvic acid	<0.0001	<0.0001	-2.1	0.16
C00490	Amino Acid	Leucine, Isoleucine and Valine Metabolism	2-Methylsuccinic acid	0.0220	0.0464	-0.7	0.59
	Amino Acid	Leucine, Isoleucine and Valine Metabolism	3-Hydroxybutanoic acid	0.0004	0.0015	-1.8	0.16
C00141	Amino Acid	Leucine, Isoleucine and Valine Metabolism	3-methyl-2-oxobutanoic acid	0.0003	0.0014	1.3	3.17
C00123	Amino Acid	Leucine, Isoleucine and Valine Metabolism	Leucine	0.0017	0.0054	-1.8	0.16
C01672	Amino Acid	Lysine Metabolism	cadaverine	<0.0001	0.0003	2.5	12.69
C00047	Amino Acid	Lysine Metabolism	Lysine	<0.0001	<0.0001	2.4	9.78
C00245	Amino Acid	Methionine, Cysteine, SAM and Taurine Metabolism	Taurine	<0.0001	<0.0001	1.6	3.97
C01481	Amino Acid	Phenylalanine and Tyrosine Metabolism	Cinnamic acid, m-hydroxy	<0.0001	<0.0001	1.7	3.95
C00483	Amino Acid	Phenylalanine and Tyrosine Metabolism	Tyramine	0.0017	0.0054	1.9	7.58
C00296	Amino acid	Phenylalanine, tyrosine and tryptophan biosynthesis	Quinic acid	<0.0001	<0.0001	-1.6	0.17
C02735	Amino Acid	Polyamine	Phenylethanolamine	0.0008	0.0030	1.5	4.44
C00134	Amino Acid	Polyamine Metabolism	Putrescine	<0.0001	0.0003	2.4	9.48
C00366	Amino Acid	Purine metabolism	Uric acid	<0.0001	<0.0001	2.1	8.22
C00078	Amino Acid	Tryptophan Metabolism	Tryptophan	0.0044	0.0116	-0.7	0.49

C05942	Amino Acid	Urea cycle; Arginine and Proline Metabolism	Pyrrole-2-carboxylic acid	<0.0001	<0.0001	-1.7	0.21
C00086	Amino Acid	Urea cycle; Arginine and Proline Metabolism	Urea	0.0061	0.0154	-2.5	0.10
	Carbohydrate	Aminosugar metabolism	Erythronic acid	<0.0001	<0.0001	-4.6	0.01
C00140	Carbohydrate	Aminosugar metabolism	N-Acetyl-glucosamine	0.0022	0.0066	2.0	8.69
C00270	Carbohydrate	Aminosugar metabolism	N-Acetylneuraminic acid	<0.0001	<0.0001	2.1	10.17
C08250	Carbohydrate	Aminosugar metabolism	Sophorose	<0.0001	<0.0001	3.9	46.96
	Carbohydrate	Aminosugar metabolism	Threonic acid	<0.0001	<0.0001	-4.0	0.03
	Carbohydrate	Fructose, Mannose and Galactose Metabolism	2-O-Glycerol-b-D-galactopyranose	0.0011	0.0039	1.2	3.34
C00095	Carbohydrate	Fructose, Mannose and Galactose Metabolism	Fructose	0.0036	0.0098	-1.6	0.32
C01582	Carbohydrate	Fructose, Mannose and Galactose Metabolism	Galactose	0.0001	0.0006	1.8	8.01
	Carbohydrate	Fructose, Mannose and Galactose Metabolism	lactose	<0.0001	<0.0001	-5.4	0.01
C00392	Carbohydrate	Fructose, Mannose and Galactose Metabolism	Mannitol	0.0006	0.0023	-1.6	0.19
C00507	Carbohydrate	Fructose, Mannose and Galactose Metabolism	Rhamnose	0.0011	0.0039	1.7	7.04
C00794	Carbohydrate	Fructose, Mannose and Galactose Metabolism	Sorbitol	<0.0001	<0.0001	-3.3	0.04
	Carbohydrate	Galactose Metabolism	Sucrose	<0.0001	<0.0001	3.5	41.83
	Carbohydrate	Glycogen Metabolism	Isomaltose	<0.0001	<0.0001	3.8	50.13
C00597	Carbohydrate	Glycolysis, Gluconeogenesis, and Pyruvate Metabolism	3-Phosphoglycerate	0.0032	0.0090	-1.0	0.38
C00258	Carbohydrate	Glycolysis, Gluconeogenesis, and Pyruvate Metabolism	Glyceric acid	0.0002	0.0008	-1.3	0.30
	Carbohydrate	Pentose Metabolism	2-deoxy-D-ribose	0.0047	0.0124	0.7	2.08
C01904	Carbohydrate	Pentose Metabolism	Arabitol	<0.0001	<0.0001	3.8	56.01
C00257	Carbohydrate	Pentose Metabolism	Gluconic acid	<0.0001	<0.0001	-2.5	0.10
C00198	Carbohydrate	Pentose Metabolism	Gluconic acid, lactone	0.0008	0.0030	1.7	4.17

C00121	Carbohydrate	Pentose Metabolism	Ribose	0.0007	0.0026	1.3	4.79
	Carbohydrate	Pentose Metabolism	Ribose-5-P	0.0139	0.0311	0.5	1.50
	Carbohydrate	Pentose Metabolism	Sedoheptulose	<0.0001	0.0002	-1.5	0.21
C00181	Carbohydrate	Pentose Metabolism	Xylose	<0.0001	<0.0001	2.1	7.10
C00738	Carbohydrate		Hexose	<0.0001	<0.0001	2.7	15.01
C02225	Energy	TCA Cycle	2-Methylcitric acid	<0.0001	<0.0001	-1.1	0.36
C00147	Energy	TCA Cycle	alpha-Ketoglutaric acid	0.0040	0.0108	-0.9	0.52
	Energy	TCA Cycle	Citric acid	<0.0001	<0.0001	-2.8	0.08
C00489	Energy	TCA Cycle	Glutaric acid	<0.0001	<0.0001	-2.2	0.11
	Energy	TCA Cycle	Glutaric acid, 2-hydroxy	<0.0001	0.0001	2.8	15.03
	Lipid	Fatty acid	2-Oxoisocaproic acid	<0.0001	0.0005	2.1	10.12
C03044	Lipid	Fatty Acid	Butane,2-3-dihydroxy	0.0004	0.0015	-0.9	0.41
C05984	Lipid	Fatty Acid	Butanoic acid, 2-hydroxy	<0.0001	<0.0001	2.7	17.30
	Lipid	Fatty Acid	Butanoic acid, 2-methyl-2-hydroxy	<0.0001	<0.0001	2.3	11.20
C04181	Lipid	Fatty Acid	Butanoic acid, 3-methyl-3-hydroxy	<0.0001	<0.0001	1.8	4.33
C01585	Lipid	Fatty acid	Hexanoic acid, 2-hydroxy	0.0075	0.0182	0.4	1.53
	Lipid	Fatty acid	Pentanoic acid, 4-methyl-2-hydroxy	<0.0001	<0.0001	3.0	19.27
C00803	Lipid	Fatty acid	Pentanoic acid, 5-amino	0.0182	0.0388	1.1	3.12
	Lipid	Fatty Alcohol	1-hexadecanoylglycerol	0.0159	0.0347	0.9	2.16
	Lipid	Fatty Alcohol	1-O-Hexadecenylglycerol	0.0069	0.0172	0.5	1.61
	Lipid	Fatty Alcohol	1-Octadecanoylglycerol	<0.0001	<0.0001	3.1	17.55
	Lipid	Fatty Alcohol	2-Propylheptanol	<0.0001	<0.0001	-2.3	0.12
	Lipid	Fatty Alcohol	Eicosanol	<0.0001	<0.0001	5.8	252.72
	Lipid	Fatty Alcohol	Heptadecanol	<0.0001	<0.0001	2.9	14.96
C00823	Lipid	Fatty Alcohol	Hexadecanol	<0.0001	<0.0001	3.6	39.41
	Lipid	Fatty Alcohol	Octadecanol	<0.0001	<0.0001	11.3	40613.14
	Lipid	Glycerolipid Metabolism	alpha-Glycerophosphorylglycerol	0.0130	0.0294	-0.5	0.70
C00116	Lipid	Glycerolipid Metabolism	Glycerol	<0.0001	<0.0001	-2.8	0.07
C00093	Lipid	Glycerolipid Metabolism	Glycerol-3-p	0.0028	0.0079	-1.0	0.35
C01177	Lipid	Inositol Metabolism	Inositol-p	0.0180	0.0387	0.8	2.20
C03365	Lipid	Inositol Metabolism	Inositol, methyl	<0.0001	<0.0001	-3.6	0.04
	Lipid	Long Chain Fatty Acid	C10:0 (Decanoic acid)	<0.0001	<0.0001	1.2	3.11
	Lipid	Long Chain Fatty Acid	C15:0 (Pentadecanoic acid)	<0.0001	<0.0001	2.4	10.93

	Lipid	Long Chain Fatty Acid	C17:0 (Heptadecanoic acid)	<0.0001	<0.0001	1.8	6.23
	Lipid	Long Chain Fatty Acid	C18:1:1 (Oleic acid)	<0.0001	0.0002	1.6	4.60
	Lipid	Long Chain Fatty Acid	C20:0 (Arachidic acid)	<0.0001	<0.0001	0.8	2.28
	Lipid	Long Chain Fatty Acid	C22:0 (Behenic acid)	0.0041	0.0110	0.8	2.09
	Lipid	Long Chain Fatty Acid	C26:0 (Cerotic acid)	0.0061	0.0154	0.8	2.35
	Lipid	Long Chain Fatty Acid	C8:0 (Caprylic acid)	<0.0001	<0.0001	-1.6	0.34
	Lipid	Long Chain Fatty Acids	C14:0 (Myristic acid)	0.0002	0.0008	0.8	1.90
C00189	Lipid	Polyamine Metabolism	Ethanolamine	<0.0001	0.0001	1.6	4.12
C02483	Lipid	Prenol Lipids	Tocopherol-y	0.0016	0.0052	-1.2	0.38
C00041	Lipid	Quinone and hydroquinone lipids	alpha-Tocopherol	0.0150	0.0330	1.2	2.80
	Lipid	Sterol	5-a-Cholestan-3-ol	0.0052	0.0135	1.0	2.50
C01444	Nucleotide	Purine metabolism	Oxamic acid	0.0019	0.0058	-2.1	0.11
C00262	Nucleotide	Purine Metabolism, (Hypo)Xanthine/Inosine containing	Hypoxanthine	0.0023	0.0067	1.6	5.20
C00385	Nucleotide	Purine Metabolism, (Hypo)Xanthine/Inosine containing	Xanthine	<0.0001	<0.0001	2.1	7.42
	Nucleotide	Purine Metabolism, Adenine containing	Adenine	<0.0001	<0.0001	1.9	7.64
C00242	Nucleotide	Purine Metabolism, Guanine containing	Guanine	<0.0001	<0.0001	3.4	29.22
C00872	Nucleotide	Pyrimidine Metabolism	Aminomalonic acid	<0.0001	0.0003	1.8	5.76
C00383	Nucleotide	Pyrimidine Metabolism	Malonic acid	<0.0001	<0.0001	1.8	5.83
C00380	Nucleotide	Pyrimidine Metabolism, Cytidine containing	Cytosine	<0.0001	<0.0001	2.9	14.91
C00178	Nucleotide	Pyrimidine Metabolism, Thymine containing	Thymine	<0.0001	0.0003	2.5	12.94
C00106	Nucleotide	Pyrimidine Metabolism, Uracil containing	Uracil	0.0003	0.0012	1.9	8.87
	Xenobiotics	Additive agent	Adipic acid	<0.0001	<0.0001	-2.7	0.07
	Xenobiotics	Benzoate Metabolism	Benzenepropanoic acid, a-hydroxy	0.0001	0.0006	1.3	2.95
	Xenobiotics	Benzoate Metabolism	Benzoic acid	<0.0001	<0.0001	0.9	2.69
C00503	Xenobiotics	Food Component/Plant	Erythritol	0.0003	0.0012	-1.0	0.41

	Xenobiotics		Diethyleneglycol	0.0012	0.0042	0.7	1.77

CT+/MG+ women vs. Uninfected women

KEGG ID	Super Pathway	Sub Pathway	Metabolite	P-value	Q-value	Coefficient	Fold Change
C01879	Amino Acid	Glutathione Metabolism	Pyroglutamic acid	0.0020	0.0060	-1.7	0.17
C00168	Amino Acid	Glycine, Serine and Threonine Metabolism	3-Hydroxypyruvic acid	0.0107	0.0253	-1.4	0.24
C00141	Amino Acid	Leucine, Isoleucine and Valine Metabolism	3-methyl-2-oxobutanoic acid	0.0177	0.0381	1.3	3.82
C01672	Amino Acid	Lysine Metabolism	Cadaverine	0.0008	0.0029	3.4	30.54
	Amino Acid	Phenylalanine and Tyrosine Metabolism	Hydrocinnamic acid	<0.0001	0.0003	1.2	3.17
	Amino Acid	Phenylalanine and Tyrosine Metabolism	p-hydroxyhydrocinnamic acid	0.0045	0.0119	2.4	55.20
C00296	Amino acid	Phenylalanine and Tyrosine Metabolism	Quinic acid	0.0002	0.0009	-1.7	0.18
C00134	Amino Acid	Polyamine Metabolism	Putrescine	0.0014	0.0048	3.1	21.69
C00366	Amino Acid	Purine metabolism	Uric acid	0.0042	0.0111	1.5	4.65
	Carbohydrate	Aminosugar metabolism	Erythronic acid	<0.0001	<0.0001	-6.2	0.00
C00645	Carbohydrate	Aminosugar metabolism	N-Acetyl mannosamine	0.0033	0.0091	0.5	0.23
C00270	Carbohydrate	Aminosugar metabolism	n-acetylneuraminic acid	0.0017	0.0055	2.4	11.11
C08250	Carbohydrate	Aminosugar metabolism	Sophorose	<0.0001	<0.0001	4.8	126.81
	Carbohydrate	Aminosugar metabolism	Threonic acid	0.0061	0.0154	-2.4	0.09
C01582	Carbohydrate	Fructose, Mannose and Galactose Metabolism	Galactose	0.0029	0.0081	2.2	9.24
	Carbohydrate	Fructose, Mannose and Galactose Metabolism	lactose	<0.0001	<0.0001	-4.1	0.02
C00507	Carbohydrate	Fructose, Mannose and Galactose Metabolism	Rhamnose	0.0125	0.0288	2.1	7.86
C00794	Carbohydrate	Fructose, Mannose and Galactose Metabolism	Sorbitol	<0.0001	<0.0001	-3.4	0.03
	Carbohydrate	Galactose Metabolism	Sucrose	<0.0001	0.0003	3.3	28.19
	Carbohydrate	Glycogen Metabolism	Isomaltose	<0.0001	0.0002	4.8	117.07

C00258	Carbohydrate	Glycolysis, Gluconeogenesis, and Pyruvate Metabolism	Glyceric acid	<0.0001	<0.0001	-2.7	0.07
	Carbohydrate	Pentose Metabolism	2-deoxy-D-ribose	0.0006	0.0025	1.4	2.32
C00257	Carbohydrate	Pentose Metabolism	Gluconic acid	<0.0001	0.0002	-2.6	0.08
	Carbohydrate	Pentose Metabolism	Sedoheptulose	0.0005	0.0020	-2.0	0.14
C00181	Carbohydrate	Pentose Metabolism	Xylose	0.0028	0.0079	1.8	5.80
C00738	Carbohydrate		Hexose	0.0016	0.0052	2.8	17.27
	Energy	TCA Cycle	Citric acid	0.0003	0.0014	-2.6	0.07
C00489	Energy	TCA Cycle	Glutaric acid	0.0015	0.0051	-2.1	0.13
	Energy	TCA Cycle	Glutaric acid, 2-hydroxy	0.0095	0.0226	2.8	15.87
	Lipid	Fatty acid	2-Oxoisocaproic acid	0.0119	0.0275	2.2	2.90
C06255	Lipid	Fatty acid	2-Oxoisovaleric acid	0.0015	0.0049	0.9	1.75
C03044	Lipid	Fatty Acid	Butane,2-3-dihydroxy	0.0050	0.0130	-1.2	0.30
C05984	Lipid	Fatty Acid	Butanoic acid, 2-hydroxy	0.0151	0.0330	2.4	11.01
	Lipid	Fatty Acid	Butanoic acid, 2-methyl-2-hydroxy	0.0119	0.0275	1.8	5.99
C04181	Lipid	Fatty Acid	Butanoic acid, 3-methyl-3-hydroxy	0.0042	0.0111	1.8	5.88
	Lipid	Fatty acid	Pentanoic acid, 4-methyl-2-hydroxy	0.0110	0.0258	2.3	14.81
C00803	Lipid	Fatty acid	Pentanoic acid, 5-amino	0.0023	0.0067	2.4	0.23
	Lipid	Fatty Alcohol	1-octadecanoylglycerol	<0.0001	<0.0001	2.7	5.66
	Lipid	Fatty Alcohol	2-Propylheptanol	0.0004	0.0017	-1.6	2.19
	Lipid	Fatty Alcohol	Eicosanol	<0.0001	<0.0001	4.3	77.23
	Lipid	Fatty Alcohol	Heptadecanol	<0.0001	<0.0001	2.3	10.21
C00823	Lipid	Fatty Alcohol	Hexadecanol	0.0010	0.0036	3.0	20.28
	Lipid	Fatty Alcohol	Octadecanol	<0.0001	<0.0001	8.9	13.60
C00093	Lipid	Glycerolipid Metabolism	Glycerol-3-p	0.0022	0.0066	-1.7	0.19
C00137	Lipid	Inositol Metabolism	Inositol	0.0059	0.0150	-1.3	0.28
C03365	Lipid	Inositol Metabolism	Inositol, methyl	<0.0001	<0.0001	-3.1	25.52
C00137	Lipid	Inositol Metabolism	Inositol, myo	0.0171	0.0371	-1.1	0.02
	Lipid	Long Chain Fatty Acid	C15:0 (Pentadecanoic acid)	<0.0001	<0.0001	2.2	9.39
	Lipid	Long Chain Fatty Acid	C17:0 (Heptadecanoic acid)	<0.0001	0.0003	1.3	3.76
	Lipid	Long Chain Fatty Acid	C18:1:1 (Oleic acid)	0.0127	0.0289	1.6	4.84
	Lipid	Long Chain Fatty Acid	C8:0 (Caprylic acid)	0.0021	0.0064	-1.8	0.17

	Lipid	Phospholipid Metabolism	O-Phosphoethanolamine	0.0011	0.0039	0.7	1099.67
C00189	Lipid	Polyamine Metabolism	Ethanolamine	0.0013	0.0043	2.0	7.39
C00041	Lipid	Quinone and hydroquinone lipids	alpha-Tocopherol	0.0173	0.0374	1.9	856.07
C00385	Nucleotide	Purine Metabolism, (Hypo)Xanthine/Inosine	Xanthine	<0.0001	0.0001	2.2	8.94
	Nucleotide	Purine Metabolism, Adenine containing	Adenine	0.0138	0.0310	1.5	6.32
	Nucleotide	Purine Metabolism, Guanine containing	2'-Deoxyguanosine	0.0012	0.0040	0.5	0.35
C00242	Nucleotide	Purine Metabolism, Guanine containing	Guanine	0.0002	0.0007	3.9	47.66
C00872	Nucleotide	Pyrimidine Metabolism	Aminomalonic acid	0.0074	0.0181	1.9	0.03
C00383	Nucleotide	Pyrimidine Metabolism	Malonic acid	0.0227	0.0471	1.2	1577148.12
C00380	Nucleotide	Pyrimidine Metabolism, Cytidine containing	Cytosine	0.0001	0.0005	3.8	43.26
C00178	Nucleotide	Pyrimidine Metabolism, Thymine containing	Thymine	0.0066	0.0165	2.7	15.18
C00106	Nucleotide	Pyrimidine Metabolism, Uracil containing	Uracil	0.0068	0.0169	2.3	10.26
	Xenobiotics	Additive agent	Adipic acid	<0.0001	0.0001	-2.0	0.95
**CT+/MG+ women vs. CT+ women**							
**KEGG ID**	**Super Pathway**	**Sub Pathway**	**Metabolite**	**P-value**	**Q-value**	**Coefficient**	**Fold Change**
	Amino Acid	Phenylalanine and Tyrosine Metabolism	Hydrocinnamic acid	0.0042	0.0150	0.8	2.33
	Amino Acid	Phenylalanine and Tyrosine Metabolism	p-Hydroxyphenylacetic acid	0.0116	0.0359	0.7	2.91
	Carbohydrate	Aminosugar metabolism	Erythronic acid	0.0164	0.0474	-1.6	0.19
C01904	Carbohydrate	Pentose Metabolism	Arabitol	0.0114	0.0355	-2.4	8.76
	Lipid	Fatty Alcohol	Eicosanol	0.0002	0.0009	-1.5	0.22
	Lipid	Fatty Alcohol	Octadecanol	<0.0001	<0.0001	-2.5	0.00
	Lipid	Long Chain Fatty Acid	C16:1 (Palmitoleic acid)	0.0147	0.0439	-1.2	0.30

Table S3| Significant metabolites associated with infection status identified by multiple linear regression and adjusted for CST

CT+ women vs. Uninfected women								
**KEGG ID**	**Super Pathway**	**Sub Pathway**	**Metabolite**	**P-value**	**Q-value**	**Coefficient**	**Fold change within CST III women**	**Fold change within CST IV women**
C00025	Amino Acid	Glutamate Metabolism	Glutamic acid	0.0001	0.0013	3.2	19.98	34.32
C00624	Amino Acid	Glutamate Metabolism	N-Acetylglutamic acid	0.0005	0.0019	1.4	3.35	3.67
C00168	Amino Acid	Glycine, Serine and Threonine Metabolism	3-Hydroxypyruvic acid	<0.0001	0.0003	-1.7	0.52	0.12
C00047	Amino Acid	Lysine Metabolism	Lysine	<0.0001	<0.0001	2.9	12.68	22.33
C00245	Amino Acid	Methionine, Cysteine, SAM and Taurine Metabolism	Taurine	<0.0001	0.0001	1.9	4.38	4.51
C01481	Amino Acid	Phenylalanine and Tyrosine Metabolism	Cinnamic acid, m-hydroxy	<0.0001	0.0004	1.8	5.95	4.85
C00082	Amino Acid	Phenylalanine and Tyrosine Metabolism	Tyrosine	0.0028	0.0019	2.0	4.55	11.27
C00296	Amino acid	Phenylalanine, tyrosine and tryptophan biosynthesis	Quinic acid	<0.0001	<0.0001	-1.8	0.15	0.18
C00366	Amino Acid	Purine metabolism	Uric acid	<0.0001	<0.0001	2.3	19.06	6.45
C05942	Amino Acid	Urea cycle; Arginine and Proline Metabolism	Pyrrole-2-carboxylic acid	0.0004	0.0019	-1.6	0.51	0.12
	Carbohydrate	Aminosugar metabolism	Erythronic acid	<0.0001	<0.0001	-4.1	0.02	0.01
C08250	Carbohydrate	Aminosugar metabolism	Sophorose	<0.0001	<0.0001	3.4	11.01	55.62
	Carbohydrate	Aminosugar metabolism	Threonic acid	<0.0001	<0.0001	-4.0	0.01	0.02
C00095	Carbohydrate	Fructose, Mannose and Galactose Metabolism	Fructose	<0.0001	0.0003	-2.6	0.01	0.36
	Carbohydrate	Fructose, Mannose and Galactose Metabolism	lactose	<0.0001	<0.0001	-5.8	0.00	0.00
C00794	Carbohydrate	Fructose, Mannose and Galactose Metabolism	Sorbitol	<0.0001	<0.0001	-3.2	0.04	0.04
	Carbohydrate	Galactose Metabolism	Sucrose	<0.0001	<0.0001	3.7	31.79	53.64

	Carbohydrate	Glycogen Metabolism	Isomaltose	0.0001	0.0013	3.1	9.48	35.35
C00597	Carbohydrate	Glycolysis, Gluconeogenesis, and Pyruvate Metabolism	3-Phosphoglycerate	0.0018	0.0019	-1.2	0.25	0.27
C00258	Carbohydrate	Glycolysis, Gluconeogenesis, and Pyruvate Metabolism	Glyceric acid	0.0025	0.0019	-1.2	0.27	0.29
C01904	Carbohydrate	Pentose Metabolism	Arabitol	<0.0001	<0.0001	3.7	80.39	30.26
C00257	Carbohydrate	Pentose Metabolism	Gluconic acid	<0.0001	0.0004	-1.7	0.18	0.11
C00181	Carbohydrate	Pentose Metabolism	Xylose	<0.0001	<0.0001	2.3	18.17	6.88
C00738	Carbohydrate		Hexose	0.0002	0.0019	2.3	11.66	8.72
C00253	Cofactors and Vitamins	Nicotinate and Nicotinamide Metabolism	Nicotinic acid	<0.0001	0.0006	-1.9	0.07	0.22
C02225	Energy	TCA Cycle	2-Methylcitric acid	<0.0001	<0.0001	-1.2	0.31	0.26
C00147	Energy	TCA Cycle	Alpha-Ketoglutaric acid	<0.0001	0.0009	-1.4	0.23	0.31
	Energy	TCA Cycle	Citric acid	<0.0001	<0.0001	-2.8	0.08	0.05
C00489	Energy	TCA Cycle	Glutaric acid	<0.0001	<0.0001	-2.5	0.05	0.13
C03044	Lipid	Fatty Acid	Butane,2-3-dihydroxy	<0.0001	<0.0001	-1.4	0.19	0.27
	Lipid	Fatty Acid	Butanoic acid, 2-methyl-2-hydroxy	0.0072	0.0019	1.2	1.02	9.91
C04181	Lipid	Fatty Acid	Butanoic acid, 3-methyl-3-hydroxy	0.0006	0.0019	1.5	10.52	2.17
	Lipid	Fatty acid	Pentanoic acid, 4-methyl-2-hydroxy	0.0039	0.0019	1.8	2.64	7.68
	Lipid	Fatty Alcohol	1-hexadecanoylglycerol	0.0017	0.0019	1.3	3.51	3.86
	Lipid	Fatty Alcohol	1-Octadecanoylglycerol	<0.0001	<0.0001	3.4	17.37	49.93
	Lipid	Fatty Alcohol	2-Propylheptanol	<0.0001	<0.0001	-2.1	0.17	0.08
	Lipid	Fatty Alcohol	Eicosanol	<0.0001	<0.0001	5.8	397.40	324.25
	Lipid	Fatty Alcohol	Heptadecanol	<0.0001	<0.0001	2.9	19.48	16.49
C00823	Lipid	Fatty Alcohol	Hexadecanol	<0.0001	<0.0001	4.1	59.36	92.59
	Lipid	Fatty Alcohol	Octadecanol	<0.0001	<0.0001	11.4	148884.00	63764.28
C00116	Lipid	Glycerolipid Metabolism	Glycerol	<0.0001	0.0011	-2.5	0.03	0.15

C03365	Lipid	Inositol Metabolism	Inositol, methyl	<0.0001	<0.0001	-3.6	0.03	0.02
	Lipid	Long Chain Fatty Acid	C10:0 (Decanoic acid)	<0.0001	0.0002	1.1	3.67	3.27
	Lipid	Long Chain Fatty Acid	C15:0 (Pentadecanoic acid)	<0.0001	<0.0001	2.2	10.36	8.56
	Lipid	Long Chain Fatty Acid	C17:0 (Heptadecanoic acid)	<0.0001	<0.0001	1.8	5.92	6.76
	Lipid	Long Chain Fatty Acid	C18:1:1 (Oleic acid)	<0.0001	0.0005	1.9	2.77	15.49
	Lipid	Long Chain Fatty Acid	C20:0 (Arachidic acid)	<0.0001	0.0003	0.9	2.07	2.91
	Lipid	Long Chain Fatty Acid	C8:0 (Caprylic acid)	<0.0001	<0.0001	-2.0	0.16	0.10
C00189	Lipid	Polyamine Metabolism	Ethanolamine	0.0006	0.0019	1.5	4.24	4.48
C12078	Lipid	Prenol Lipids	Dehydroabietic acid	0.0055	0.0019	-0.5	0.43	0.87
C02483	Lipid	Prenol Lipids	Tocopherol-y	0.0008	0.0019	-1.4	0.26	0.17
C01444	Nucleotide	Purine metabolism	Oxamic acid	0.0069	0.0019	-2.1	0.10	0.20
C00262	Nucleotide	Purine Metabolism, (Hypo)Xanthine/Inosine containing	Hypoxanthine	0.0084	0.0019	1.6	7.90	3.74
C00385	Nucleotide	Purine Metabolism, (Hypo)Xanthine/Inosine containing	Xanthine	<0.0001	<0.0001	1.6	3.64	7.72
	Nucleotide	Purine Metabolism, Adenine containing	Adenine	0.0002	0.0019	1.6	4.10	6.46
C00242	Nucleotide	Purine Metabolism, Guanine containing	Guanine	<0.0001	0.0002	3.1	18.66	27.29
C00872	Nucleotide	Pyrimidine Metabolism	Aminomalonic acid	<0.0001	0.0011	2.0	8.45	7.36
C00383	Nucleotide	Pyrimidine Metabolism	Malonic acid	<0.0001	0.0001	1.7	6.58	5.80
C00380	Nucleotide	Pyrimidine Metabolism, Cytidine containing	Cytosine	<0.0001	0.0005	2.8	23.83	9.66
	Xenobiotics	Additive agent	Adipic acid	<0.0001	<0.0001	-2.3	0.07	0.13
	Xenobiotics	Benzoate Metabolism	Benzenepropanoic acid, a-hydroxy	0.0013	0.0019	1.2	1.60	5.10
	Xenobiotics	Benzoate Metabolism	Benzoic acid	<0.0001	0.0012	0.9	4.31	1.95
C00503	Xenobiotics	Food Component/Plant	Erythritol	0.0010	0.0019	-1.0	0.38	0.36
	Xenobiotics		Diethyleneglycol	0.0020	0.0019	0.7	3.00	1.90
**CT+/MG+ women vs. Uninfected women**								

KEGG ID	Super Pathway	Sub Pathway	Metabolite	P-value	Q-value	Coefficient	Fold change within CST III women	Fold change within CST IV women
C00025	Amino Acid	Glutamate Metabolism	Glutamic acid	0.0019	0.0133	3.9	26.23	77.28
	Amino Acid	Phenylalanine and Tyrosine Metabolism	Hydrocinnamic acid	0.0011	0.0089	1.0	1.00	4.20
C00296	Amino acid	Phenylalanine, tyrosine and tryptophan biosynthesis	Quinic acid	0.0002	0.0018	-1.9	0.15	0.16
C00366	Amino Acid	Purine metabolism	Uric acid	0.0011	0.0084	1.9	9.67	4.75
	Carbohydrate	Aminosugar metabolism	Erythronic acid	<0.0001	<0.0004	-5.6	0.06	0.00
C08250	Carbohydrate	Aminosugar metabolism	Sophorose	<0.0001	0.0009	4.3	391.99	57.63
	Carbohydrate	Fructose, Mannose and Galactose Metabolism	lactose	<0.0001	<0.0006	-4.6	0.03	0.01
C00794	Carbohydrate	Fructose, Mannose and Galactose Metabolism	Sorbitol	<0.0001	0.0005	-3.3	0.19	0.02
	Carbohydrate	Galactose Metabolism	Sucrose	<0.0001	0.0009	3.6	106.91	30.64
	Carbohydrate	Glycogen Metabolism	Isomaltose	0.0013	0.0099	4.0	733.37	28.00
C00258	Carbohydrate	Glycolysis, Gluconeogenesis, and Pyruvate Metabolism	Glyceric acid	<0.0001	0.0010	-2.5	0.34	0.05
C00257	Carbohydrate	Pentose Metabolism	Gluconic acid	0.0095	0.0498	-1.6	0.22	0.15
C00181	Carbohydrate	Pentose Metabolism	Xylose	0.0015	0.0113	2.0	15.48	5.11
C00147	Energy	TCA Cycle	Alpha-Ketoglutaric acid	0.0089	0.0470	-1.4	0.52	0.23
	Energy	TCA Cycle	Citric acid	0.0008	0.0067	-2.7	0.20	0.04
C00489	Energy	TCA Cycle	Glutaric acid	0.0004	0.0037	-2.4	0.12	0.10
C03044	Lipid	Fatty Acid	Butane,2-3-dihydroxy	0.0001	0.0016	-1.7	0.19	0.18
	Lipid	Fatty Alcohol	1-octadecanoylglycerol	<0.0001	<0.0007	3.0	29.64	22.21
	Lipid	Fatty Alcohol	2-Propylheptanol	0.0038	0.0240	-1.4	0.37	0.17
	Lipid	Fatty Alcohol	Eicosanol	<0.0001	<0.0002	4.4	46.99	88.43
	Lipid	Fatty Alcohol	Heptadecanol	<0.0001	<0.0003	2.3	8.12	10.87
C00823	Lipid	Fatty Alcohol	Hexadecanol	0.0003	0.0034	3.6	81.44	35.82
	Lipid	Fatty Alcohol	Octadecanol	<0.0001	<0.0001	8.9	4203.70	7608.50

C03365	Lipid	Inositol Metabolism	Inositol, methyl	<0.0001	<0.0005	-3.1	0.11	0.03
	Lipid	Long Chain Fatty Acid	C15:0 (Pentadecanoic acid)	<0.0001	<0.0008	2.0	6.24	7.64
	Lipid	Long Chain Fatty Acid	C17:0 (Heptadecanoic acid)	<0.0001	0.0010	1.4	4.34	3.95
	Lipid	Long Chain Fatty Acid	C18:1:1 (Oleic acid)	0.0050	0.0304	1.9	4.99	11.71
	Lipid	Long Chain Fatty Acid	C8:0 (Caprylic acid)	0.0003	0.0031	-2.2	0.14	0.08
	Lipid	Phospholipid Metabolism	O-Phosphoethanolamine	0.0045	0.0274	0.7	1.40	2.35
C00189	Lipid	Polyamine Metabolism	Ethanolamine	0.0061	0.0354	1.8	10.87	5.25
C00385	Nucleotide	Purine Metabolism, (Hypo)Xanthine/Inosine containing	Xanthine	0.0028	0.0185	1.5	2.50	7.31
	Nucleotide	Purine Metabolism, Guanine containing	2'-Deoxyguanosine	0.0085	0.0453	0.5	1.00	2.01
C00242	Nucleotide	Purine Metabolism, Guanine containing	Guanine	0.0013	0.0099	3.5	87.39	27.95
C00872	Nucleotide	Pyrimidine Metabolism	Aminomalonic acid	0.0066	0.0376	2.1	2.49	12.09
C00380	Nucleotide	Pyrimidine Metabolism, Cytidine containing	Cytosine	0.0004	0.0035	3.8	1528.40	10.40
	Xenobiotics	Additive agent	Adipic acid	0.0009	0.0075	-1.6	0.28	0.21

CT+/MG+ women vs. CT+ women

KEGG ID	Super Pathway	Sub Pathway	Metabolite	P-value	Q-value	Coefficient	Fold change within CST III women	Fold change within CST IV women
	Amino Acid	Phenylalanine and Tyrosine Metabolism	Hydrocinnamic acid	0.0071	0.0498	0.80	1.00	2.69
	Lipid	Fatty Alcohol	Eicosanol	0.0002	0.0030	-1.50	0.12	0.27
	Lipid	Fatty Alcohol	Octadecanol	<0.0001	<0.0001	-2.47	0.03	0.12

Table S4. PERMANOVA Assessing Relationship of Biological, Demographic and Behavioral factors with the Vaginal Metabolome

	DF	Sum of Squares	R2	F	P-value
**Community State Type**	4.00	1.98	0.11	5.03	<0.0001
**Infection Status**	2.00	2.21	0.12	11.18	<0.0001
**Age**	4.00	0.47	0.03	1.18	0.27
**No. Sex Partners**	4.00	0.33	0.02	0.83	0.64
**Condom use**	3.00	0.30	0.02	1.03	0.40
**Pelvic Inflammatory Disease**	1.00	0.06	0.00	0.65	0.64
**HIV Status**	2.00	0.63	0.03	3.17	0.01
**Residual**	124.00	12.23	0.67		
**Total**	144.00	18.21	1.00		

Table S5 | Metabolites detected in Amies controls

Metabolite	Average Relative Concentration / 200uL	Standard Error of Mean (200uL)
2-Methylmalic acid	21.58	0.78
3,4-Dihydroxybutanoic acid	2.90	0.12
Alanine	145.99	6.46
Benzoic acid, 2-methyl	86.74	0.95
butanoic acid, 4-hydroxy	1.08	0.06
C16:0	149.35	16.05
C18:0	68.50	6.76
Fumaric acid	3.84	0.17
Glucose	1.79	0.13
Glycolic acid	524.22	11.18
glyoxylic acid	49.49	2.17
lactic acid	1945.43	85.72
malic acid	1.33	0.10
Malic acid	2009.40	34.17
Oxalic acid	17.24	1.27
Pentanoic acid, 3-methyl-2-hydroxy	4.27	0.49
phosphoric acid	31046.09	1097.94
pyrophosphate (4:1)	2.81	0.30
pyruvic acid	10117.92	518.51
Succinic acid	14.71	1.78

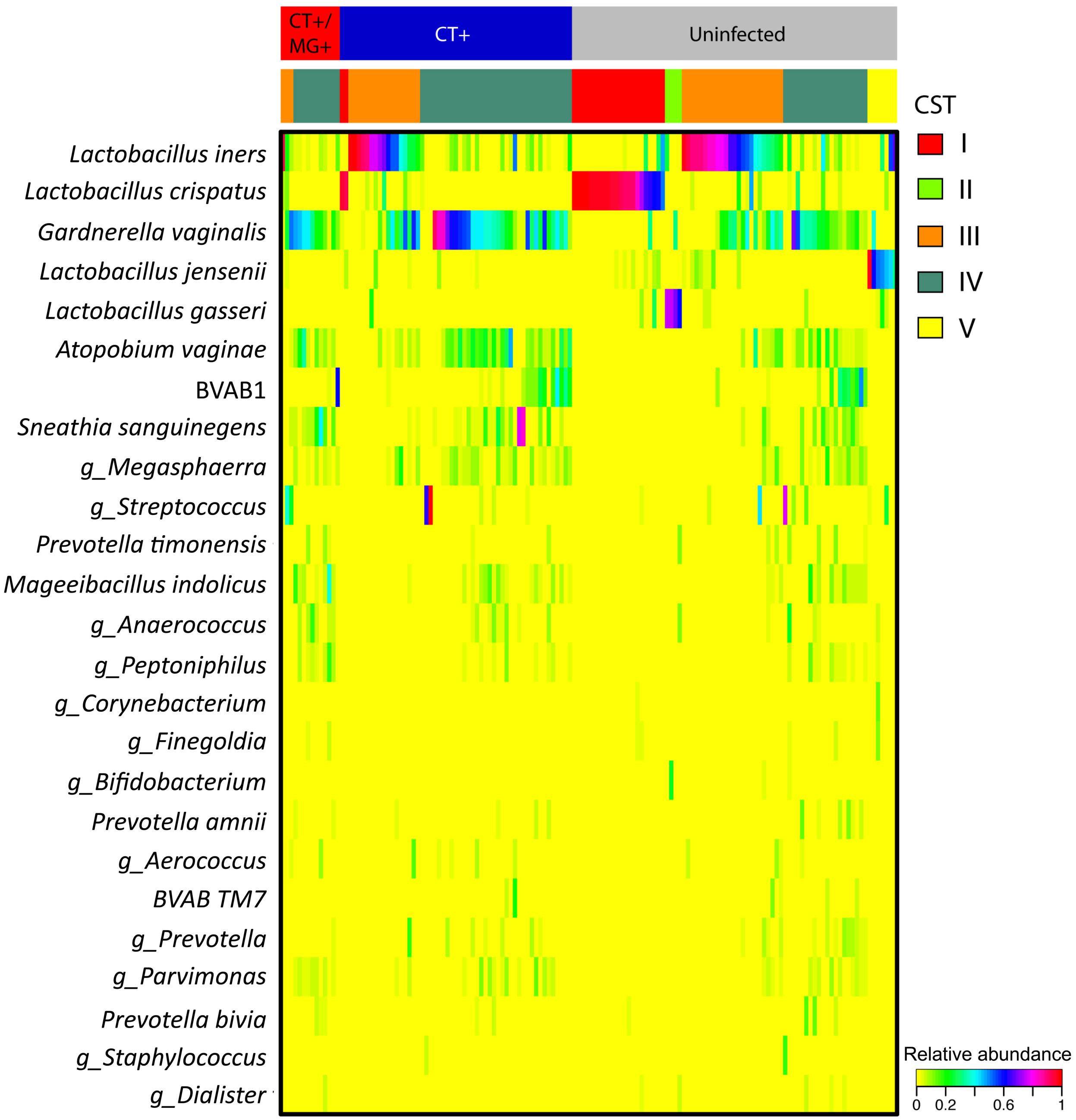

Figure S1 | Heatmap displaying the relative abundance of the 25 most abundant taxa in samples from MG+/CT+ co-infected, CT+ mono-infected, and uninfected women. Community state types (CSTs) are indicated in the second row from the top.
